# Dual Energy CT Pulmonary Angiography with 6g Iodine—A Propensity Score-Matched Study

**DOI:** 10.1371/journal.pone.0167214

**Published:** 2016-12-01

**Authors:** Andreas Meier, Kai Higashigaito, Katharina Martini, Moritz Wurnig, Burkhardt Seifert, Dagmar Keller, Thomas Frauenfelder, Hatem Alkadhi

**Affiliations:** 1 Institute of Diagnostic and Interventional Radiology, University Hospital Zurich, University of Zurich, Zurich, Switzerland; 2 Department of Biostatistics; Epidemiology, Biostatistics and Prevention Institute, University of Zurich, Zurich, Switzerland; 3 Interdisciplinary Emergency Department, University Hospital Zurich, University of Zurich, Zurich, Switzerland; Universite de Bretagne Occidentale, FRANCE

## Abstract

**Objective:**

To evaluate the performance of low contrast media (CM) dose dual-energy computed tomography pulmonary angiography (CTPA) with advanced monoenergetic reconstructions in patients with suspected pulmonary embolism (PE).

**Materials and Methods:**

The study had institutional review board approval; all patients gave written informed consent. Forty-one patients (25 men, 16 women, mean age 62.9±14.7 years) undergoing low CM dose (15ml, 6g iodine) dual-energy CTPA with advanced monoenergetic reconstructions were matched via propensity-scoring based on logistic regression analysis with a comparison group of 41 patients (24 men, 17 women, mean age 62.7±13.9 years) undergoing standard CM dose single-energy CTPA (80ml, 24g iodine). Subjective (noise, artifacts) and objective (attenuation, noise, contrast-to-noise ratio (CNR)) image quality was assessed by two blinded, independent readers. All patients underwent clinical follow-up after three months for evaluation of adverse events.

**Results:**

Interrater agreement for subjective image quality in both groups ranged from fair to excellent (ICC: 0.46–0.84); agreement for objective image quality was excellent (ICC: 0.83–0.93). There was no significant difference regarding subjective noise (p = 0.15–0.72) and artifacts (p = 0.16–1) between the low and the standard CM dose group. There was no significant difference regarding CNR between the CM dose groups (p = 0.11–0.87). Seven of the 41 (17%) patients in the low and 5/41 (12%) in the standard CM dose group were diagnosed with PE (p = 0.32). No patient suffered from subsequent PE or PE-associated death during the follow-up period.

**Conclusion:**

Dual-energy CTPA with advanced monoenergetic reconstruction is feasible with 6g iodine and allows for the diagnosis and safe exclusion of central, lobar, and segmental PE.

## Introduction

Pulmonary embolism (PE) represents an emergency condition with an incidence of 60-70/100’000 cases per year and a high associated mortality [1, 2]. Mortality is substantially higher in patients with coexisting nephropathy [3], a common comorbidity in this patient population as shown in the PIOPED II study where 19% of patients were excluded from contrast enhanced computed tomography (CT) due to elevated serum creatinine levels [4].

The imaging method of choice for the diagnosis and exclusion of PE is CT pulmonary angiography (CTPA), as it is widely available and has a high accuracy [5]. CTPA requires the intravenous administration of iodinated contrast media (CM), which has a potential nephrotoxic effect particularly in patients with impaired kidney function who may develop contrast-induced nephropathy (CIN) [6].

The causality of CIN after CM administration is controversially discussed in the literature. Several studies have shown that the risk of developing CIN is related to the volume of intravenously administered iodinated CM [7, 8], and that there is no dose threshold below which the administration of CM can be considered safe [9]. In distinction, other studies found that CIN is only a risk factor in patients with elevated serum creatinine levels or even suggest this phenomenon to be coincidental and unrelated to the administration of iodinated CM [10, 11]. Still, the European Society or Urogenital Radiology (ESUR) currently recommends using the lowest possible amount of CM allowing for a definite diagnosis in patients with impaired renal function or other associated risk factors [12]. Yet another reason for reducing CM is the fact that iodine absorbs more ionizing radiation, which results in an increased patient dose and hence, in a significant increase in the amount of DNA double-strand breaks [13].

In the early era of multislice helical CT, a typical CTPA protocol required the administration of 120 to 150 mL CM, equaling 30–42 g iodine [14–16]. Since then, several advances in CT technology enabled the reduction of the amount of CM to 22–23 g iodine [17, 18]. The introduction of conventional dual-energy CTPA showed potential for a further reduction of the iodine load to 15–20 g [19]. A recently introduced algorithm in dual-energy CT called advanced virtual monochromatic image reconstruction combines the advantages of high attenuation at low energy levels with lower image noise at higher energy levels [20, 21]. In a preliminary study in patients undergoing CTPA with 60 ml (18 g iodine), use of this algorithm resulted in a 1.8-fold increase of the contrast-to-noise ratio (CNR) compared to conventional monoenergetic image reconstructions [22], suggesting potential for further CM volume reduction. However, decreasing acquisition times in combination with the use of low-kilovoltage protocols in state of the art CT angiography require optimized CM injection protocols in order to achieve optimal image quality [23–25].

The purpose of this study was to evaluate the performance of low CM dose dual-energy CTPA with advanced monoenergetic reconstructions in patients with suspected PE. Our hypothesis was that this algorithm would enable sufficient opacification of the pulmonary arteries for the diagnosis and safe exclusion of PE with an iodine load of only 6 g.

## Materials and Methods

### Patients

This study—including both a retrospective and a prospective part—had institutional review board and local ethics committee approval (Kantonale Ethikkommission Zürich). Written informed consent was obtained from all patients in both study parts.

**Fig 1:**
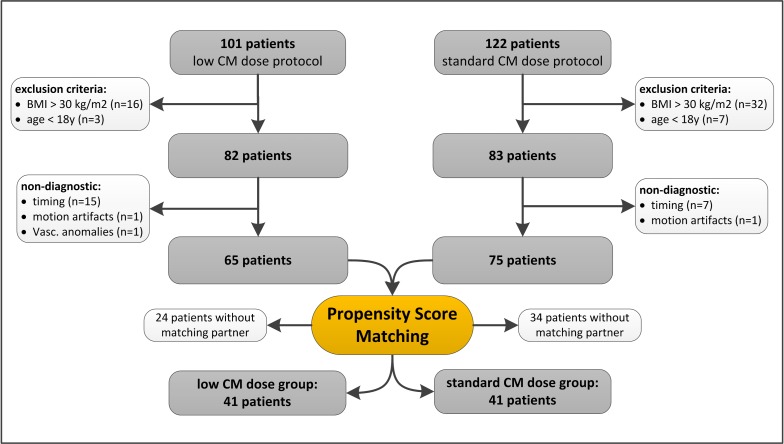
Patient selection pathway and propensity score matching.

For the prospective, low CM dose group, a total of 101 in- and outpatients were screened for inclusion over a 6-month period (from May to October 2015). The indication for CT in all patients was clinically suspected PE. Exclusion criteria were severely impaired renal function (defined as estimated glomerular filtration rate (eGFR) below 30 ml/min/1.73m^2^) (n = 0), hypersensitivity to iodinated CM (n = 0), a body mass index (BMI) above 30 kg/m^2^ (n = 16), or patients younger than 18 years (n = 3). The reason for excluding patients with a BMI>30 mg/m^2^ is the fact that dual-energy CT images in obese patients may be associated with excessive noise [26]. Thus, 82/101 patients (81%) were included in the prospective study part and underwent CTPA with a low CM dose. From those 82 patients, one patient (1%) had a non-diagnostic CTPA examination due to motion artifacts, one patient (1%) due to vascular anomalies, and 15 patients (18%) had a non-diagnostic examination due to insufficient contrast in the pulmonary arteries. In summary, 17 patients (21%) had non-diagnostic imaging and were therefore excluded from further analysis. Finally, a total of 65 patients were included in the low CM dose group (**Fig 1**).

The decision whether an examination was of diagnostic image quality or not was made by the study conductor and the attending radiologist assessing each CTPA examination in consensus. Visual criteria were a sufficient opacification of the pulmonary arteries from the pulmonary trunk to the segmental level allowing to diagnose or to rule-out PE with a high degree of confidence. If an examination was considered non-diagnostic, it was repeated by applying our standard CM dose protocol with an amount reduced by 15 ml CM in order to not to exceed the regularly administered standard dose of 80 ml, and patients were excluded from further analysis.

The prospective, low CM dose population was compared to another group of patients, which we retrospectively included into this study. Here, 122 in- and outpatients were included over a 9-month period (from August 2014 to April 2015). These patients were examined on the same CT scanner but with our standard CM dose protocol. We applied equal exclusion criteria to the retrospective study group: severely impaired renal function (n = 0), hypersensitivity to iodinated CM (n = 0), a BMI above 30 kg/m^2^ (n = 32), and age under 18 years (n = 7). Patients with a BMI above 30 kg/m^2^ were similarly excluded to obtain comparable study populations. 83/122 patients (68%) fulfilled these inclusion criteria. Eight of these 83 patients (9.6%) had a non-diagnostic examination, seven (8.4%) were excluded due to insufficient opacification of the pulmonary arteries and one (1.2%) due to severe motion artifacts. Thus, a total of 75 patients were finally included in the standard CM dose group (**Fig 1**).

We generated a propensity score (see statistical analysis section below), to match patients one to one from each CM dose group. Patients without a matching partner were excluded as previously shown [27]. By doing so, a total of 82 patients from both groups remained in the study for comparison: 41 patients in the low CM dose group (25 men, 16 women; mean age men 63.8±13.9 years, range 26–83 years; mean age women 61.5±16.3 years, range 27–89 years), and 41 patients in the standard CM dose group (24 men, 17 women; mean age men 64.8±13.3 years, range 42–88 years; mean age women 59.6±14.6 years, range 28–83 years). Twenty of the 82 patients (24%) had an impaired renal function, defined as an eGFR between 30 and 60ml/min/1.73m^2^.

### Clinical information and follow-up

Clinical and demographic data of all patients were extracted from the electronic patient records. Laboratory information was extracted likewise, including eGFR data within one day prior to CT.

Since we evaluated CTPA with a low CM dose, hereby putting the patient to a potential risk of missing PE in case of non-sufficient opacification of the pulmonary arteries, we assessed the validity of a negative CTPE scan in both CM dose groups by using a three-month clinical follow-up strategy as previously shown [28, 29]. For this, we evaluated the occurrence of subsequent PE or PE-related deaths within 3 months after the initial CTPA examination. Most patients had follow-up examinations at our hospital addressing this question and therefore the information was retrieved from their electronic records. If there was no in-house follow-up available, the patient’s referring physician was contacted to exclude PE-associated events.

### CT data acquisition and contrast media protocol

All patients underwent CTPA on a third generation dual-source CT scanner (SOMATOM Force, Siemens Medical Systems, Forchheim, Germany): the low CM dose group with dual-energy (90/150SnkVp), the standard CM dose group with single-energy (100 kVp) with further data acquisition parameters as shown in the **S1 Table**. The scanning direction was cranio-caudal during mid-inspiratory breath-hold for approximately 10 seconds. Data acquisition was timed with bolus tracking using a trigger set in the pulmonary trunk. In both groups, we used a monophasic protocol and the CM was injected with a power injector (CT Exprés, Swiss Medical Care, Lausanne, Switzerland) in an antecubital vein. Prior to CM injection, vessel patency was tested with a saline flush of 10 ml. The CM injection was followed by another saline flush in both groups for keeping the bolus compact and for reducing residual CM in brachial and central venous vessels.

In the standard CM dose group, we used our routine PE protocol and injected 80 ml (iodine load 24 g) CM (Imeron 300, 300 mg J/ml, Bracco S.p.A, Milan, Italy) at 4 ml/sec. Current literature advocates for flow rates in CTPA not below 1.2 g iodine / second [30]. Though our reconstruction algorithm results in an almost twofold CNR increase, we reduced the flow rate to 0.6 g iodine / second in the low CM dose group. This allows prolonging the injection time to 10 seconds which makes the protocol less vulnerable in terms of bolus timing. In order to compensate for the slower flow rate we increased the iodine concentration which results in a higher peak enhancement, which is most desirable for an optimal arterial enhancement [31]. Following this, we injected 15 ml (iodine load 6 g) of a high concentrated CM (Imeron 400, 400 mg J/ml, Bracco S.p.A, Milan, Italy) at 1.5 ml/sec (see **S1 Table**). This low CM dose protocol was tested and validated in a small preliminary group of 10 patients, which were not included into the actual study. By analyzing the bolus tracking protocols in these 10 patients, we found 30 Hounsfield Units (HU) as the best threshold for initiating the scan start.

### CT image reconstruction

From each patient in the low CM dose group, advanced virtual monoenergetic images were reconstructed at 40 keV using a dedicated application (Monoenergetic Plus Application Class) and software (syngo.viaVA30, Siemens) because it has been demonstrated that image reconstructions at 40 keV improve the contrast of dual-energy CTPA [22]. All images from both groups were reconstructed with a slice thickness of 2 mm and an increment of 1.6 mm, using advanced modeled iterative reconstruction (ADMIRE, strength level 3) and a medium soft tissue convolution kernel (Bf40).

### CT data analysis

All image data were sent to the local picture archive and communication system (PACS) (Impax 6.5.5; Agfa HealthCare, Mortsel, Belgium) and were read on dedicated monitors (Barco nio 3mp LED, Barco N.V., Kortrijk Belgium).

*Subjective image quality* concerning overall noise and perivascular artifacts of the pulmonary trunk (PT), the right lower lobe pulmonary artery (RLLPA), the right inferior pulmonary vein (RIPV), and the descending aorta as well as the superior vena cava (SVC) and the brachiocephalic vein (BC) (on the side where the contrast media was administered) were assessed in each dataset by two blinded, independent readers ([xx] and [yy] (blinded for review)), with 6 and 4 years of experience in chest radiology) by using a four-point Likert scale as previously shown [32]: A score of 0 represented complete or almost complete absence of either noise / artifacts, a score of 1 represented mild noise / artifacts, a score of 2 represented moderate noise / artifacts, and a score of 3 represented marked noise / artifacts. Each image series was read with fixed window settings (width 600 HU; level 150 HU).

*Objective image quality* was assessed by the same two readers ([xx] and [yy]) and as previously shown [22]: Attenuation (in HU) was measured in the PT, RLLPA, RIPV, and in the descending aorta at the level of the left ventricle by placing a circular region of interest (ROI) adapted to the vessel size in the center of the vessel. Furthermore, attenuation was measured in the periscapular musculature in a homogeneous area, and the standard deviation of attenuation was used as an indicator for image noise. CNR was calculated for each vessel as follows:

$$CNR=\frac{attenuation\;vessel\;[HU]–attenuation\;muscle\;[HU]}{image\;noise\;[HU]}$$

### Statistical analysis

**Fig 2:**
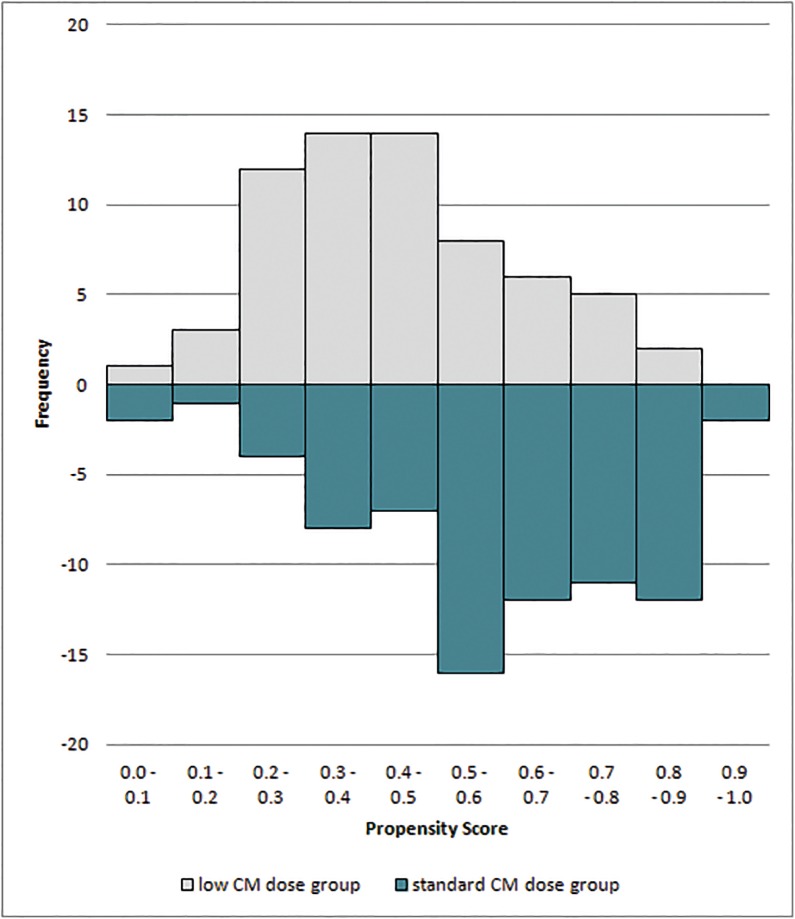
Distribution of propensity scores in the study population.

To limit the observational character of the study when including both a prospective and a retrospective study population and for reducing the effect of potential confounding, we performed a propensity-score matching for the two study groups. For the computation of the propensity score, the following variables were included into a logistic regression model: age, gender, BMI, eGFR, Wells score, presence or absence of PE, dyspnea, chest pain, and reduced general state of health (**Table 1**). The validity of logistic regression was assessed using the Hosmer-Lemeshow test. After generating the propensity score, patients were matched one to one with a caliper of 0.2 on the logit scale using the package Matching in R (R Foundation for Statistical Computing, Vienna, Austria). Patients without a matching partner were excluded (**Fig 2**).

**Table 1:** Propensity score-matched patient characteristics.

Patient characteristic	Low CM dose group (n = 41)	Standard CM dose group (n = 41)	p-values
Sex ratio (men/women)	25 / 16	24 / 17	.82
Age (year) ^#^	62.9 ± 14.7	62.7 ± 13.9	.95
Body mass index (kg/m^2^) ^#^	24.3 ± 3.3	24.3 ± 4.1	.81
eGFR (ml/min) ^#^	81.7 ± 20.0	77.3 ± 29.2	.48
Wells-Score ^#^	1.1 ± 1.2	1.0 ± 1.1	.58
Dyspnea (yes/no)	14/27	13/28	.82
Chest pain (yes/no)	14/27	13/28	.82
Reduced general state of health (yes/no)	8/33	8/33	1.0
Pulmonary embolism (yes)	7/34	5/36	.54

^#^Data is expressed as mean ± standard deviation.

eGFR = estimated glomerular filtration rate.

Interrater agreement for both subjective and objective image quality was assessed using the intra-class correlation coefficient (ICC). Values were interpreted as follows: <0.40, poor agreement; 0.40–0.59, fair agreement; 0.60–0.74, good agreement; and >0.75, excellent agreement (15). Comparisons regarding artifacts and subjective noise at the above mentioned locations between the low and standard CM group were performed using the Fisher's exact test. To test for differences between objective measures in the low and the standard CM group Mann-Whitney U test or—in normally distributed data—unpaired Student's t-test were used. Occurrences of PE in the two groups were compared using the χ^2^-test. All two-tailed p-values below 0.05 were considered statistically significant. All analyses (except of the propensity score-matching) were performed using the statistical software package SPSS (release 22.0 IBM SPSS Statistics, Armonk, NY: IBM Corp.).

## Results

### Radiation dose

Median volume CT dose index (CTDI_vol_) was 5.9 mGy in the low CM dose group and 5.6 mGy in the SD dose group, with no significant differences between groups (p = 0.12–0.96). Median dose-length product was 206 mGy*cm and effective dose was 2.9 mSv, and the median size specific dose estimate was 7.5 mGy in the low CM dose group and 7.0 mGy in the standard CM dose group.

### Subjective image quality

Interrater agreement for subjective noise and artifacts ranged from fair to excellent (ICC 0.46–0.84) for both groups and for each location.

**Fig 3:**
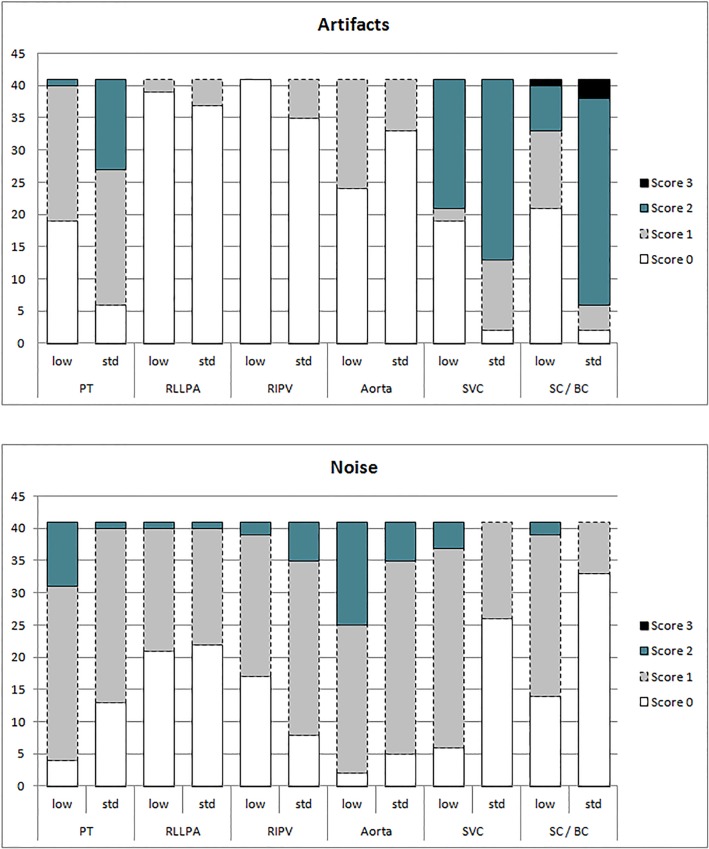
Distribution of artifacts (**a**) and subjective noise (**b**) between the low and the standard CM dose group. (low = low CM dose group, std = standard CM dose group, PT = pulmonary trunk, RLLPA = right lower lobe pulmonary artery, RIPV = right inferior pulmonary vein, SVC = superior vena cava, SC = subclavian vein, BC = brachiocephalic vein)

Subjective image quality was good with both readers rating noise and artifacts as being either absent (score 0) or mild (score 1), and rarely moderate (score 2) for each location in both subgroups except of artifacts in the standard CM dose group in the SVC and brachiocephalic / subclavian vein and in the low CM dose group in the SVC. Here, artifacts were predominantly moderate (score 2) or marked (score 3) due to undiluted contrast media (**Fig 3**).

**Fig 4:**
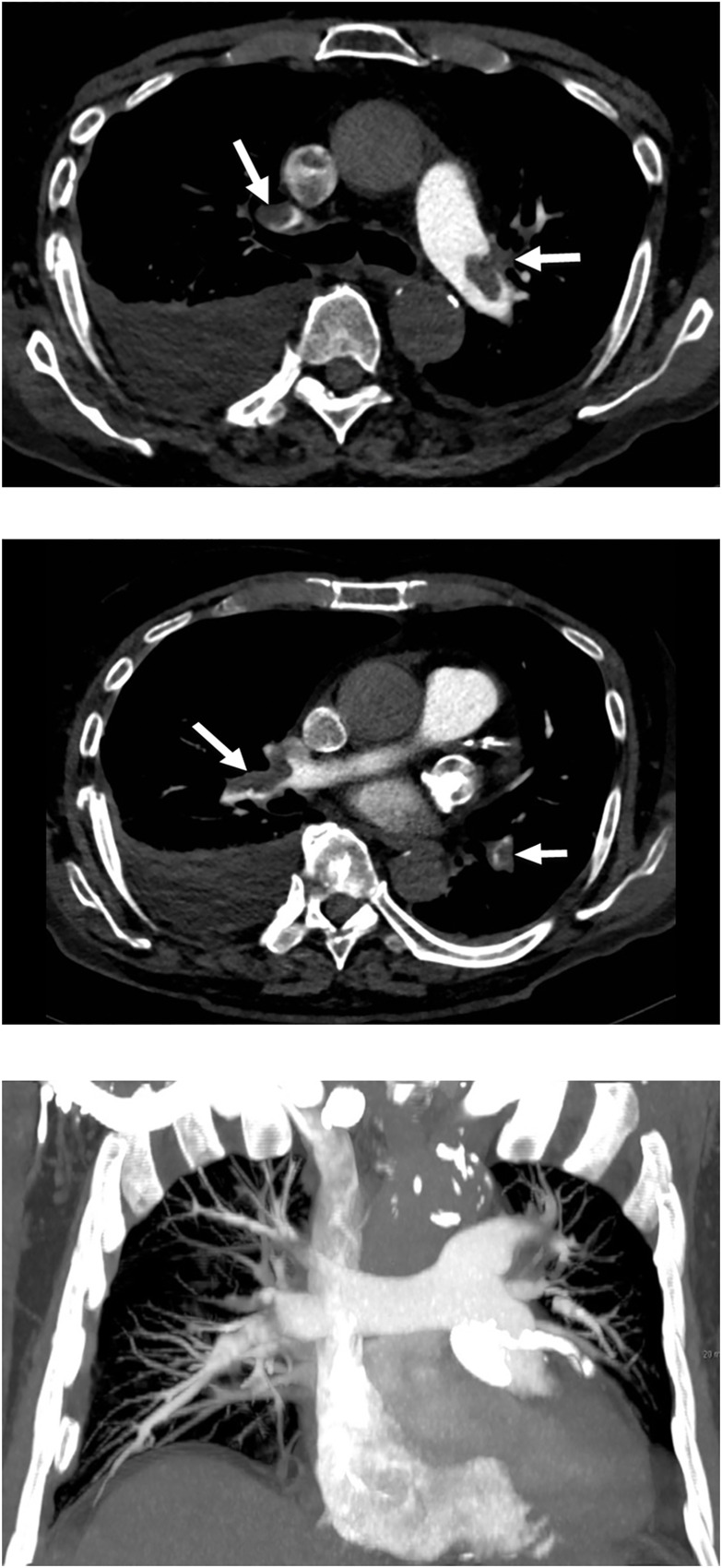
55-year-old male patient with dyspnea. Dual-energy CTPA with advanced monoenergetic image reconstructions and administration of 6 g iodine shows good opacification of the pulmonary vasculature with emboli in the right and left pulmonary arteries (arrows) (**a**) and in both lower lobe arteries (arrows) (**b**). Coronal thick (30 mm) maximum intensity projection (MIP) reformation (**c**) demonstrates good opacification of the pulmonary artery system to the segmental level.

**Fig 5:**
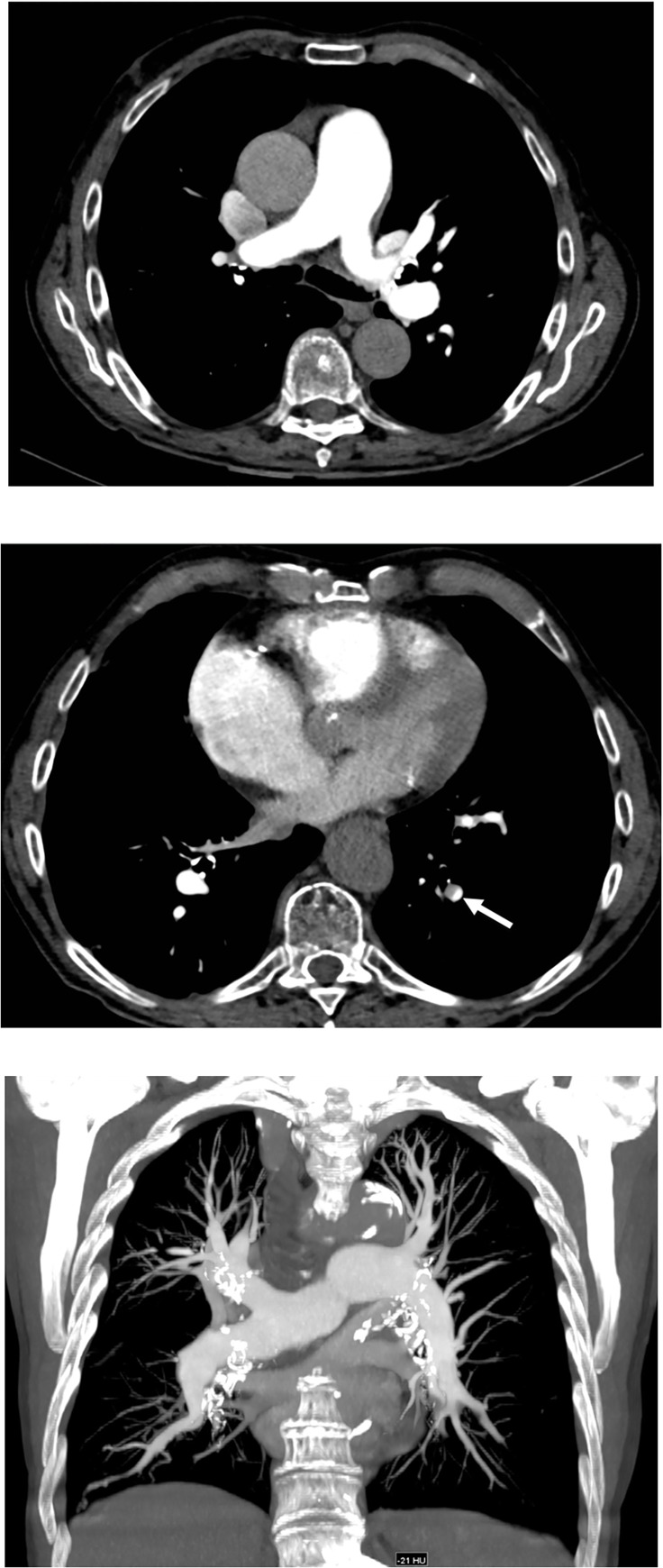
63-year-old male patient with chest pain and dyspnea. Dual-energy CTPA with advanced monoenergetic image reconstructions and administration of 6 g iodine shows good opacification of the main pulmonary trunk and arteries (**a**) and a clot at the segmental level of the left lower lobe (arrow) (**b**). Coronal thick (30 mm) maximum intensity projection (MIP) reformation (**c**) demonstrates good opacification of the pulmonary artery system to the segmental level.

There were no significant differences between the low and the standard CM dose group regarding subjective noise (low CM dose group: mean score 0.5±0.5 to 1.3±0.5; standard CM dose group: 0.2±0.4 to 1.0±0.5, p = 0.15–0.72). There were also no significant differences between groups regarding artifacts (low CM dose group: 0±0 to 0.7±0.8; standard CM dose group: 0.1±0.3 to 1.8±0.7, p = 0.16–1). Representative image examples of patients from the low CM dose group are provided in **Figs 4** and **5**.

### Objective image quality

**Fig 6:**
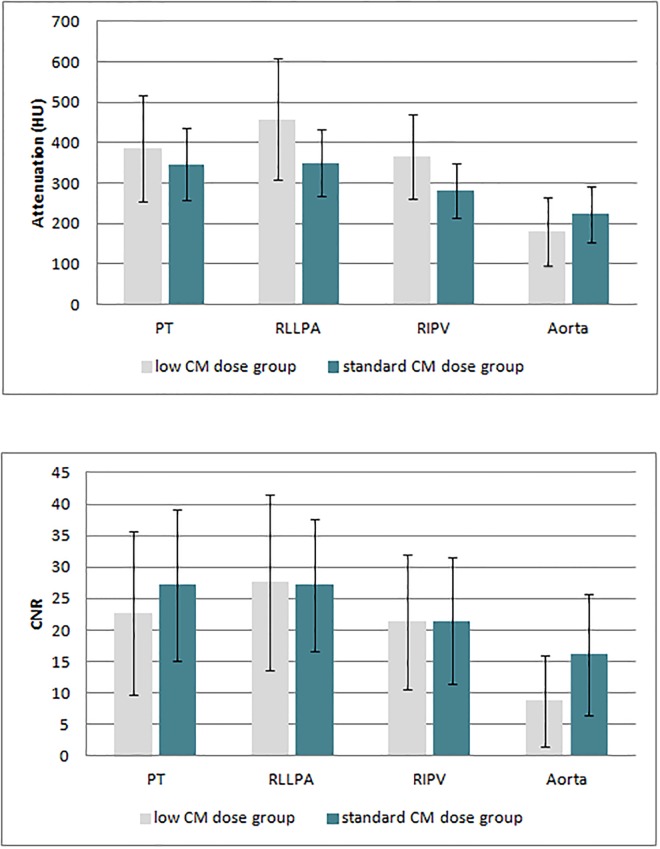
Mean values and standard deviation of attenuation (**a**) and contrast-to-noise ratio (CNR, **b**) at the different locations. (CM = contrast media, PT = pulmonary trunk, RLLPA = right lower lobe pulmonary artery, RIPV = right inferior pulmonary vein).

Interrater agreement for attenuation, noise and CNR was excellent in both groups (ICC 0.83–0.93). Attenuation was equal in both groups at the level of the PT (p = 0.2) and higher in the low compared to the standard CM dose group in the RLLPA and RIPV (both p = 0.001). Attenuation in the aorta was higher in the standard CM dose group, however, without reaching statistical significance (p = 0.051) (**Fig 6A**).

Mean noise in the low CM group was 16±3 HU (range 10–21 HU) compared to 11±3 HU (range 7–22 HU) in the standard CM dose group, being not significantly different (p = 0.59).

CNR was slightly higher in the standard CM dose group in the PT and the aorta without being statistically significant (both p = 0.11) whereas CNR in the RLLPA as well as in the RIPV were similar for both groups (p = 0.87 and 0.84) (**Table 2**, **Fig 6B**).

**Table 2:** CNR in the low and in the standard CM dose group.

	Low CM dose group	Standard CM dose group	p-values
**CNR PT**	22.8 ± 13.0	27.2 ± 12.0	p = 0.11
**CNR RLLPA**	27.6 ± 14.0	27.1 ± 10.4	p = 0.87
**CNR RIPV**	21.7 ± 10.5	21.4 ± 10.0	p = 0.84
**CNR aorta**	8.8 ± 7.3	16.1 ± 9.7	p = 0.11

CM = contrast media. CNR = contrast-to-noise ratio. PT = pulmonary trunk. RLLPA = right lower lobe pulmonary artery. RIPV = right inferior pulmonary vein.

### Clinical follow-up

In total, 12/82 patients (14%) were diagnosed with PE: 7/41 (17%) in the low CM dose group and 5/41 (12%) in the standard CM dose group, with no difference between groups (p = 0.32). Also, clinical information at three months showed that no patient suffered from subsequent PE or PE-associated death during the follow-up period.

## Discussion

Impaired renal function represents a clinically relevant comorbidity among patients with suspicion of PE. In our study population, 24% of patients had a reduced eGFR (ranging between 30 and 60 ml/min/1.73m^2^) and 22% were 70 years or older, both risk factors for developing CIN [8]. Since the risk of CIN is related to the dose of administered CM [7, 8], these patients benefit from an examination with a reduced CM dose. By using advanced monoenergetic reconstructions from dual-energy CT, we showed that in patients with a BMI below 30 kg/m^2^, the iodine load for CTPA can be considerably reduced to 6 g, while the subjective and objective image quality of the examination remains preserved. Most importantly, CTPA with the low CM dose safely excluded PE in our low pretest probability population, as shown in the clinical follow-up period of three months.

In general, a reduction of CM dose is often paralleled by a higher rate of non-diagnostic examinations due to suboptimal vessel opacification. In this study, non-diagnostic scans in the standard CM dose group occurred in 8% of patients, being in line with data reported in the literature [33]. In the low CM dose group, the frequency of non-diagnostic CTPA scans due to suboptimal vessel opacification was higher (18%). The most probable reason for this is the small CM bolus with a low peak enhancement and short plateau phase requiring a short scan window. This is also reflected by the lower CNR in the vessels proximally and distally to the pulmonary arteries and veins in the low as compared to the standard CM dose group found in this study. Hence, optimal bolus timing is pivotal for a diagnostic examination with low CM volumes and is more difficult to realize compared to a CM protocol with higher amounts of CM [34].

Additional complicating factors are the variability of cardiac output and the Valsalva maneuver resulting in a transient interruption of contrast potentially impacting on the opacification of pulmonary arteries [35]. It is assumed that low CM dose protocols are more susceptible to these factors as compared to those with a standard CM dose.

Besides the administration of CM, the exposure of patients to ionizing radiation with its theoretical risk represents a continuous concern. Several techniques are available for achieving a marked radiation dose reduction, including tube current modulation, tube voltage adaptation and use of iterative image reconstruction techniques [36]. Using several of these techniques, we achieved in our study population a considerably lower radiation dose exposure (median CTDI_vol_ 5.6–5.9 mGy in both groups, respectively) to that previously published using the same CT scanner technology (CTDI_vol_ 8.1 mGy) and a population with similar BMI (24.3 kg/m^2^ in our study compared to 25 kg/m^2^ in [37]. Of course, repetition of non-diagnostic CTPA examinations in the low CM dose group resulted in a higher radiation exposure as well as a higher CM dose in this subgroup of patients. It is necessary to point out though that the total amount of CM in patients with repeated CTPA did not exceed but was that normally administered in our standard CM dose protocol. Nevertheless, the application of the low CM dose CTPA protocol shown here must be carefully evaluated before scanning.

In case of successful clinical implication with non-diagnostic rates comparable to standard CM dose protocols, our approach might be particularly interesting for elderly patients with suspected PE and concomitant kidney disease, focusing on preservation of the remaining kidney function rather than on radiation dose saving.

In this feasibility study, we focused on central, lobar and segmental pulmonary arteries but did not specifically assess subsegmental pulmonary arteries. The rational behind this is the fact that–although current CT technology allows for making the diagnosis of subsegmental PE–these imaging findings do not affect the patients’ outcome. Thus, peripheral embolic disease in subsegmental arteries does not necessarily need treatment but has been implied with overdiagnosis having the adverse effect of overtreatment and unnecessary radiation exposure [38].

This study had several limitations. First, the study population used for comparison was enrolled retrospectively. We addressed this issue by using propensity score-matching for minimizing confounding. Second, to our knowledge the applied image reconstruction algorithm of dual-energy CT data is currently offered by one vendor only, and generalizability of our results to other CT scanners is thus limited. Third, we excluded patients with a BMI>30 kg/m^2^, because previous studies reported impaired image quality because of high noise in dual-energy CT of obese patients, caused by limited photon penetration of the low voltage tube [26]. However, it might be possible that third generation dual-source CT being characterized by a higher tube power [39] could provide dual-energy images with sufficient quality also at a higher BMI. Fourth, despite of blinding the readers could have noticed the type of image reconstruction and hence, CM group during their qualitative and quantitative image read-out. This is related to the fact the visual impression of dual-energy CT data sets differ from that of standard reconstructions. Finally, our sample size in both groups is relatively small, and future studies should include more patients in randomized, prospective trials to prove the validity of our results.

In conclusion, our study shows that with optimal CM bolus timing, CTPA with dual-energy data acquisition in combination with advanced monoenergetic image reconstruction is feasible with a low iodine dose of 6 g allowing for the diagnosis and safe exclusion of central, lobar and segmental PE. This could be particularly useful in patients with coexisting kidney disease in which preservation of residual kidney function is mandatory.

## Supporting Information

S1 Table CT scanning and contrast media protocols (DOCX)
