# Correction: Dual Energy CT Pulmonary Angiography with 6g Iodine—A Propensity Score-Matched Study

**DOI:** 10.1371/journal.pone.0172251

**Published:** 2017-02-09

**Authors:** Andreas Meier, Kai Higashigaito, Katharina Martini, Moritz Wurnig, Burkhardt Seifert, Dagmar I. Keller, Thomas Frauenfelder, Hatem Alkadhi

The sixth author’s name is incorrect in the published article. The correct name is: Dagmar I. Keller. The correct citation is: Meier A, Higashigaito K, Martini K, Wurnig M, Seifert B, Keller DI, et al. (2016) Dual Energy CT Pulmonary Angiography with 6g Iodine—A Propensity Score-Matched Study. PLoS ONE 11(12): e0167214. doi:10.1371/journal.pone.0167214
