# Supplementary material for: Dual Energy CT Pulmonary Angiography with 6g Iodine—A Propensity Score-Matched Study
Source: PLoS One. 2016 Dec 1;11(12):e0167214. doi: 10.1371/journal.pone.0167214 (PMC5132396; doi:10.1371/journal.pone.0167214)
Supplement: S1 Table — (DOCX) [file pone.0167214.s001.docx]

**Supporting Information Table S1:** CT scanning and contrast media protocols

|  | **Low CM dose group** | **Standard CM dose group** |
| --- | --- | --- |
| **Scanning parameters** |  | |
| Number of x-ray sources | 2 | 1 |
| Tube voltage (kVp) | 90 (tube A) /  150 (tube B°) | 100 |
| Qual. reference tube current-time product (effective mAs) | 153 (tube A) /  85 (tube B) | 84 |
| Tube current modulation | On | on |
| Rotation time (sec) | 0.25 | 0.50 |
| Pitch | 0.55 | 1.20 |
| Collimation (mm) | 0.6 | 0.6 |
| **Injection parameters** |  | |
| Iodine concentration (mg/ml) | 400 | 300 |
| Injection phase I: contrast media (ml)  Injection phase II: NaCl (ml) | 15  50 | 80  50 |
| Flow rate phase I (mL/sec)  Flow rate phase II (mL/sec) | 1.5  4.0 | 4.0  4.0 |
| Total iodine load (g) | 6 | 24 |
| Bolus tracking threshold (region of interest in pulmonary trunk) (HU) | 30 | 100 |
| Delay to scan start (sec) | 4 | 10 |
| **Reconstruction parameters** |  | |
| Section thickness (mm) | 2 mm | 2 mm |
| Kernel | Bf 40 | Bf 40 |
| **Radiation dose parameters** |  | |
| Volume CT dose index (mGy)^#^ | 5.9 ± 1.0 | 5.6 ± 2.1 (p=0.33) |
| Dose-length product (mGy x cm)^#^ | 206 ± 43 | 206 ± 72 (p=0.96) |
| Effective dose (mSv) ^#^ | 2.9 ± 0.6 | 2.9 ± 1.0 (p=0.96) |
| Size-specific Dose Estimates (mGy)^#^ | 7.5 ± 0.9 | 7.0 ± 2.1 (p=0.12) |

°tube B with tin filtration.

^#^data is expressed as mean ± standard deviation.
